# Travel-Related Tick-Borne Encephalitis, Israel, 2006–2014

**DOI:** 10.3201/eid2301.160888

**Published:** 2017-01

**Authors:** Eyal Meltzer, Yael Paran, Yaniv Lustig, Shmuel Stienlauf, Miriam Weinberger, Eli Schwartz

**Affiliations:** Chaim Sheba Medical Center, Tel Hashomer, Israel (E. Meltzer‚ Y. Lustig, S. Stienlauf, E. Schwartz);; Tel Aviv University, Tel Aviv, Israel (E. Meltzer‚ S. Stienlauf, M. Weinberger, E. Schwartz);; Tel Aviv Sourasky Medical Center, Tel Aviv (Y. Paran); Assaf Harofeh Medical Center, Zerifin, Israel (M. Weinberger)

**Keywords:** Tick-borne encephalitis, travel, Israel, arboviruses, ticks, vector-borne infections, viruses, zoonoses

## Abstract

During 2006–2014, four tick-borne encephalitis (TBE) cases occurred among Israeli travelers. We calculated TBE incidence at 321.0, 45.0, 13.2, and 7.5 cases/100,000 travelers/year of travel to Sweden, Switzerland, Austria, and Germany, respectively. TBE incidence among travelers to these destinations appears to justify TBE vaccination in accordance with World Health Organization recommendations.

Tick-borne encephalitis (TBE), an arboviral zoonosis transmitted mostly through the bite of *Ixodes* spp. ticks, is endemic to many popular tourist destinations in Europe (*1*). TBE vaccine is considered immunogenic and safe. It is highly effective in reducing TBE in disease-endemic areas such as Austria, the first country to include the vaccine in its national vaccine program. The World Health Organization (WHO), the US Centers for Disease Control and Prevention, and the Israeli Ministry of Health have published advice on TBE vaccination for travelers (*2*–*4*), suggesting limiting vaccination to travelers planning activities with high-risk exposures. However, the evidence base for these recommendations is not well established.

The most comprehensive study of travel-related TBE (*5*) comprised 38 travel-related cases of TBE and estimated an annual attack rate of 1 case/1.3–2 × 10^6^ travelers. However, the lack of an accurate denominator and absence of data on exposure time raise questions about its accuracy. We investigated the epidemiology of TBE in travelers from Israel and assessed the risk for travel-related TBE.

## The Study

In Israel, viral encephalitis is a notifiable disease. Diagnostic tests for TBE in Israel have been available since 2006 and are performed only in the Israeli Central Virology Reference Laboratories (Tel Hashomer, Israel) of the Israeli Ministry of Health by using an indirect immunofluorescence commercial kit (Euroimmun AG, Lübeck, Germany) according to manufacturer instructions.

As numerator, we included all confirmed cases of TBE (according to criteria adopted by the European Commission in 2012 [6]). During 2006–2014, a total of 4 TBE cases were diagnosed in Israel: 1 case each acquired in Austria, Russia, and Sweden and 1 case acquired in either Germany or in Switzerland (Table 1; Technical Appendix).

**Table 1:** Clinical, epidemiologic, and laboratory data for TBE cases, Israel*

Variable	Case-patient 1	Case-patient 2	Case-patient 3	Case-patient 4
Destination	Austria	Germany, Switzerland	Sweden	Russia
Probable area of exposure	Salzburgland	Baden-Wurttemberg	Northwest of Stockholm	Southwestern Siberia
Month of travel	2014 Jun	2012 Sep	2011 May–Aug	2010 Aug
Duration of travel, d	3	14	107	17
Duration of probable exposure to tick habitat	1 h	4 d	Undetermined	17
Recorded tick bite	Yes	No	No	No
Neurologic manifestations during acute phase	Diplopia → stupor, aphasia, quadriparesis	Dysphagia, dysgeusia, bilateral facial nerve paralysis	Meningismus, mild confusion, dysarthria → Lt facial nerve. paralysis	Acute confusion, stupor
Neurologic outcome at 6 mo	Complete motor recovery, difficulty in complex tasks, depression.	Complete recovery	Complete recovery	Complete recovery
TBE serology results†				
First serum sample	IgM positive, IgG positive	IgM positive, IgG negative	IgM positive, IgG positive	IgM positive, IgG positive
Convalescent-phase serum sample	IgM positive, IgG 10-fold increase	IgM positive, IgG seroconversion	IgM positive‡, IgG positive‡	ND

*ND, not done; TBE, tickborne encephalitis. †West Nile virus was ruled out serologically in all cases. ‡Direct comparison between serum samples was not possible because initial sample was taken abroad.

As denominator we used 2 sets of data. For the first, in emulation of Steffen et al. (*5*), we calculated TBE attack rate per 100,000 traveler entries during the study period, according to the number of Israeli tourists obtained from the United Nations World Tourism Organization database (Table 2). Cumulatively, 3,928,164 Israeli travelers had entered these countries during the study period. The combined attack rate for these countries (according to whether Germany or Switzerland was considered the place of acquisition of 1 case) ranged from 1 case per 837,528 to 1 case per 573,493 Israeli tourist entries.

**Table 2:** Calculated incidence of travel-related TBE for selected countries, Europe*

Study (source country, years of study [reference])	Country of travel	Population studied	TBE incidence		
			Cases/tourist entries	Cases/person-weeks	Cases/100,000 travel-years
Incidence of TBE disease					
This study (Israel, 2006–2014)	Germany	Travelers	1/1,634,192	1/697,700	7.5
	Austria	Travelers	1/732,160	1/393,556	13.2
	Switzerland	Travelers	1/ 578,052	1/199,102	45.0
	Sweden	Travelers	1/128,642	1/16,270	321.0
	Russia	Travelers	1/855,118	NA	NA
Reusken et al. (Netherlands [*7*])	Austria	Travelers	NA	1/1,380,952	3.8
Incidence of TBE seroconversion					
Sanchez et al. (USA [*8*])	Bosnia-Herzegovina	Military personnel	NA	1/12,501	416.0, 8.3†
McNeil et al. (USA [*9*])	Germany	Military personnel	NA	1/4,775	1,088.9, 21.8†

*NA, not available; TBE, tick-borne encephalitis. †Estimated incidence of clinical disease if 98% of infections are subclinical.

Because the United Nations World Tourism Organization database lacks information about duration of stay, we also used data on the absolute number of nights stayed by Israeli travelers during the study period from the published tourism statistics of Switzerland, Germany, and Austria (*10*–*12*) (similar data for Russia were lacking); for Sweden, we obtained the number of overnight stays from Statistics Sweden (E. Meltzer, unpub. data). TBE incidence ranged from 1 case per 697,700 person-weeks (Germany) to 1 case per 16,270 person-weeks (Sweden), which is equivalent to an annual incidence of 7.5–321.0 cases/100,000 travelers per year of travel (Table 2).

## Conclusions

TBE is endemic to some of the most popular tourist destinations in developed countries, raising questions about vaccination and advice to travelers. However, there is such a dearth of published research on the actual risk to travelers that TBE is termed a neglected disease in travel medicine (*13*). A 2016 study of travel-related TBE estimated an attack rate of 1 case per 1.3–2 × 10^6^ tourist-entries, concluding that vaccination should be offered only to travelers planning activities resulting in at-risk exposures (*5*). By using travel duration data, we found that the actual TBE incidence rate in travelers from Israel to Sweden, Austria, Germany, and Switzerland (Table 2) is at least as high as that of the local population (5/100,000 in Austria and <1/100,000 in Germany, for example [*1*]) and is higher than the WHO-recommended threshold for universal TBE vaccination (an annual incidence >5/100,000 population) (*14*).

Few other studies on the incidence of travel-related TBE have been published. These studies also suggest a substantial TBE risk to travelers that is similar in range to our results (Table 2).

Our study has several limitations. Calculating incidence rates when event numbers are small runs the risk for overestimation, according to the law of small numbers. However, by considering the total period of TBE test availability in Israel (9 years), rather than just the years in which cases were detected, we believe we avoided such overestimation. In addition, reporting of notifiable diseases might be incomplete, and clinicians’ awareness of the need to consider TBE in a returning traveler from Europe or northern Asia probably is insufficient. Also, milder TBE cases might have been overlooked. These considerations also might have resulted in an underdetection of cases. Statistics on overnight stays might not include some travelers, such as expatriates in private residences or those staying with families. On the other hand, in such travel scenarios, persons with TBE might have been treated abroad and therefore missed. We believe the combined effect of these limitations is more likely to have led to an underestimation than an overestimation of TBE risk for travelers. Indeed, our calculations included all Israeli tourists traveling in all seasons, whereas as shown in the 4 cases reported here as well (Table 1), TBE risk is seasonal: were only summer tourists considered, incidence rates of travel-related TBE probably would have been even higher.

It is interesting to compare the data presented here on TBE to another travel-related, vaccine-preventable flavivirus: Japanese encephalitis (JE). The risk for JE among travelers is considered low: <1/1 × 10^6^ travelers staying 1 month (which equals an incidence lower than 1.2 cases/100,000 travelers/year of travel) (*2*). Our findings, as well as those of previous reports, suggest that the risk posed by TBE far exceeds that posed by JE to travelers. Only 1 case of JE in an Israeli traveler has been reported in >2 decades (*15*), whereas at least 4 TBE cases were diagnosed during the past 9 years. Similar to JE, TBE had been documented to cause severe and fatal illness among travelers, but whereas JE vaccines for travelers are promoted by most government advisory boards in developed countries, very little is done regarding TBE vaccination. This difference probably underlies the fact that in Israel, for example, during 2012–2014, a total of 46,773 JE vaccine doses were distributed, whereas only 960 TBE vaccine doses were sold during the same period (E. Schwartz, unpub. data).

In conclusion, actual incidence of TBE among Israeli travelers to Austria, Germany, Sweden, and Switzerland appears to be higher than the threshold recommended by WHO for universal TBE vaccination. Vaccination should be recommended to all travelers with potential exposure to tick habitat in these destinations. However, TBE vaccine appears to be greatly underutilized among Israeli travelers.

Technical Appendix Clinical and epidemiologic information about tick-borne encephalitis in travelers returning to Israel.
