# Supplementary material for: Travel-Related Tick-Borne Encephalitis, Israel, 2006–2014
Source: Emerg Infect Dis. 2017 Jan;23(1):119–21. doi: 10.3201/eid2301.160888 (PMC5176226; doi:10.3201/eid2301.160888)
Supplement: Technical Appendix — Clinical and epidemiologic information about tick-borne encephalitis in travelers returning to Israel. [file 16-0888-Techapp-s1.pdf]

Article DOI: http://dx.doi.org/10.3201/eid2301.160888

Travel-Related Tick-Borne Encephalitis, Israel

Technical Appendix

Clinical and Epidemiologic Information about Tick-borne Encephalitis in Travelers Returning to Israel

Case-patient 1: A 25-year-old previously healthy Israeli woman had traveled on June 2014 for 3 days to Austria, where she resided near Salzburg. The only time she had spent outdoors was a short hike for <1 hour in a wooded area, where she recalled sitting barefoot in a forest clearing. The same night she removed 2 ticks from behind her right knee. A week later, fever, severe headache, and vomiting developed. After 2 days, diplopia developed; physical examination revealed nuchal rigidity and paralysis of the fourth cranial nerve. Lumbar puncture showed mixed lyphocytic-neutrophilic pleocytosis and elevated protein. Within a day, stupor, aphasia, and intermittent strabismus developed. In the next few days, her fever resolved and consciousness improved but she remained quadriparetic. After 2.5 months of rehabilitation, there was still left hand monoparesis and some cognitive and mental impairment. On follow-up after 6 months, she still had impaired concentration and decreased ability to study and was treated for depression.

Case-patient 2: A 26-year-old previously healthy Israeli man had traveled to Germany and Switzerland in September 2012. His itinerary began with 4 days of hiking and cycling in rural areas in Baden-Württemberg; his later travel in the Alps at various heights (both above and below 1,500 m) was motorized, with less exposure to wooded areas. Two weeks after his return fever, diarrhea, and cough developed. After a week his fever resolved; however, mouth numbness, difficulty eating solid food, and a change in taste developed. Physical examination showed bilateral facial nerve paralysis. Lumbar puncture revealed borderline abnormalities in cerebrospinal fluid. serology showed seroconversion to tickborne encephalitis (TBE) and was

negative for Lyme disease and for West Nile virus (WNV). He was treated with physiotherapy 4 months later was fully recovered.

Case-patient 3: A 39-year-old previously healthy Israeli man had resided since May 2011 for 4 months in a village northwest of Stockholm, Sweden. A month before his return to Israel, he had onset of low fever, which resolved within 3 weeks. However, 10 days later, fever recurred, accompanied by difficulty in speech and some degree of confusion. He was hospitalized in Sweden: a lumbar puncture revealed lymphocytic pleocytosis. After a few days, his general condition improved. On his return, right peripheral facial nerve palsy was present on physical examination. Blood serology for TBE IgG and IgM was positive in Sweden, as well as in Israel. Serology for WNV and Lyme disease were negative. He was treated with physiotherapy for 4 months and fully recovered after 3 months.

Case-patient 4: A 15-year-old previously healthy Israeli boy had traveled to Russia for 17 days in August 2010. He spent 14 days in south-central Siberia hiking, and 3 more days in Moscow. Seven days after returning he had onset of fever and vomiting. A day after fever onset, he became confused to a point that he could not recognize his family members and had upper limb cramps. Neurologic examination showed confusion and drowsiness; the physical examination was otherwise normal. Lumbar puncture showed mild mixed lymphocytic and neutrophilic pleocytosis, elevated protein, and low glucose. Brain magnetic resonance imaging showed minimal thickening of the cortex in the temporal-occipital and subinsular areas. Serology for TBE was positive for IgG and IgM. Over the next few days, his neurologic status improved, and a follow-up visit a month later showed complete resolution of all neurologic symptoms without sequelae.
